# A case of *Strongyloides* hyperinfection associated with tuberculosis

**DOI:** 10.1186/2052-0492-1-7

**Published:** 2013-11-26

**Authors:** Yoshiaki Iwashita, Kei Suzuki, Asami Masui, Eiji Kawamoto, Kazuto Yokoyama, Akitaka Yamamoto, Yukinari Omori, Ken Ishikura, Tsuyoshi Hatada, Masaki Fujioka, Taichi Takeda, Hiroshi Imai

**Affiliations:** Emergency and Critical Care Center, Mie University hospital, 2-147 Edobashi, Tsu, Mie, Japan

**Keywords:** Strongyloidiasis, Hyperinfection, Ivermectin, Tuberculosis, Cellular immunity

## Abstract

Strongyloidiasis is a parasitic infection that occurs in tropical regions. Hyperinfection, which is an accelerated autoinfection, is often associated with an immunosuppressive state, such as HTLV-1 infection or steroid use. Immunosuppression can also lead to reactivation of tuberculosis infection. These infections may have interacted as a result of impaired cellular immunity. A 28-year-old Nepali male was referred to our hospital for slight abdominal pain and high fever. An abdominal CT scan showed ascites and intestinal swelling. He was admitted with suspected gastroenteritis. Results of stool microscopy on the third day of hospitalization revealed abundant strongylid larvae. We diagnosed a *Strongyloides* hyperinfection and prescribed ivermectin. Although the numbers of strongylid organisms in the patient’s stool soon diminished, his temperature remained high. After receiving a second dose of ivermectin on day 17, he was transferred to a nearby hospital for observation, where he was noted to have massive pleural effusion. He returned to our hospital where his pleural effusion was found to be positive for adenosine deaminase (ADA), and he was diagnosed with a tuberculosis infection. *Strongyloides* hyperinfection can occur in a non-endemic region. It can be associated with tuberculosis infection possibly due to impaired cellular immunity. It is important to consider other possible infections when treating a patient with an infection associated with impaired cellular immunity.

## Background

Strongyloidiasis is a parasitic infection rarely seen in mainland Japan. Hyperinfection of this organism sometimes leads to fatal outcome because hyperinfections often occur in patients whose cellular immunity is impaired. Here, we present a case involving a 28-year-old Nepali male suffering from a hyperinfection of *Strongyloides* whom we successfully treated with ivermectin. After the strongyloidiasis was treated, the patient was found to have tuberculosis. This case may suggest a relationship between strongyloidiasis and tuberculosis attributable to impaired cellular immunity.

## Case presentation

A 28-year-old Nepali male was referred to our hospital with suspected gastroenteritis. He had a 3-week history of high fever (around 38°C-39°C), appetite loss, and general fatigue. He had visited a nearby hospital, where he was found, on abdominal CT, to have ascites and duodenal swelling without free air. On admission to our hospital, he complained of general fatigue and slight abdominal pain but was not experiencing diarrhea. He had been in Japan for 4 years and had not been back to Nepal for the last 2 years. He was living with four other Nepali males who were not experiencing similar physical problems. He denied being males who sex with males. He was a Hindu and ate Japanese food bought in a nearby supermarket. He had no remarkable past medical history and was not taking any medications. He works so hard on a supermarket in Japan and he could not sleep well. He also feels loss of appetite. He had been repeated low-grade fever around 37°C and general fatigue before coming to our hospital.

In general, the patient did not look sick. His height was 170 cm, weight was 46.5 kg, and BMI was 16.1. His blood pressure was 90/64, pulse rate 109/min, and body temperature 36.2°C. His eyes were not anemic or icteric, and chest sounds were clear. His abdomen was soft and flat, although there was slight tenderness in the left lower quadrant.

**Figure 1:**
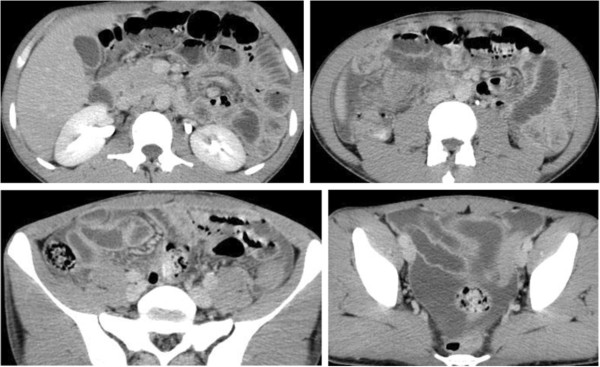
**Abdominal CT scan on admission.** Ascites and intestinal swelling can be seen.

Laboratory data on admission showed a slightly elevated white blood cell count of 7,600/μl (neutrophil 5,920/μl, lymphocyte 880/μl, monocyte 790/μl, eosinophil 0/μl, and basophil 10/μl) and highly elevated CRP (13.9 mg/dl). His albumin was low at 3.0 g/dl. A chest radiograph was clear, and there were no signs of pneumonia. An abdominal computed tomography (CT) scan on admission showed ascites with swollen intestines but no free air (Figure 1).

**Figure 2:**
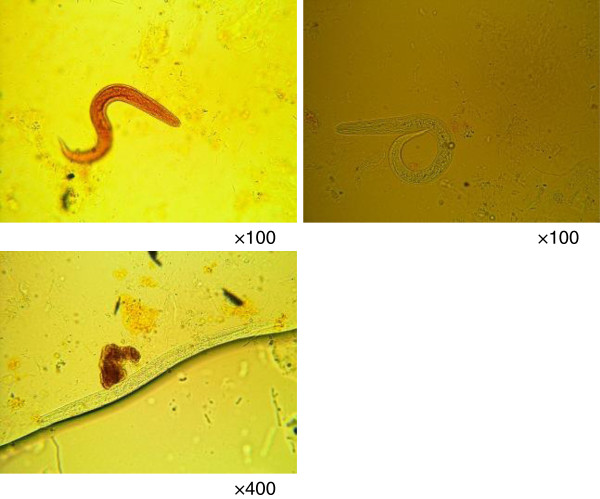
**Stool microscopy.** Massive strongylids can be seen in the stool sample.

**Figure 3:**
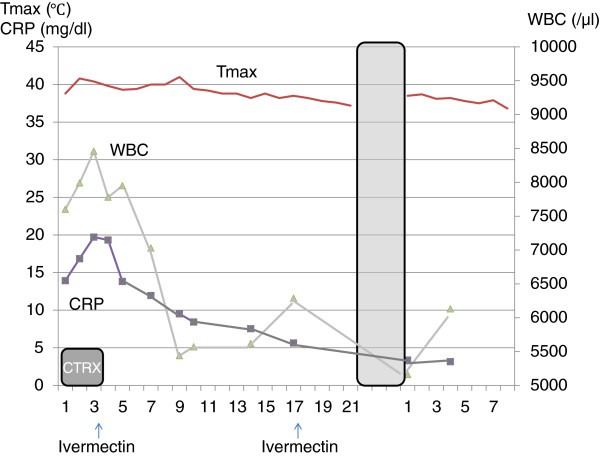
**Hematology and clinical chemistry data.** Although the patient’s markers of inflammation decreased after ivermectin administration, his temperature remained high.

We hospitalized the patient for observation. His temperature rose to 40°C, and laboratory data on day 2 showed highly elevated inflammatory markers. Microbiological studies including sputum, urine, and blood cultures were all negative. He was constipated until day 3. His stool on day 3 looked normal; it was not watery and contained no blood. Stool microscopy revealed numerous strongylid larvae (Figure 2). We diagnosed a *Strongyloides* hyperinfection. Serum human T-cell leukemia virus type 1 (HTLV-1) and human immunodeficiency virus (HIV) tests were negative, and CD4 counts were not decreased. We prescribed 12 mg of ivermectin. Although his general condition improved within 24 h after treatment in that he was eating well and had no diarrhea or abdominal pain, his temperature continued to exceed 39°C. The course of his inflammatory laboratory data and temperature are shown in Figure 3. On day 7, his stool sample was negative for strongylid larvae. An abdominal CT scan on day 6 revealed massive ascites, but the culture of the ascites on day 9 was negative for strongylid larvae. He did not complain of dyspnea throughout his hospitalization. A second 12-mg dose of ivermectin was administered on day 17.

After the second dose of ivermectin, the patient still had a low-grade fever of 37°C-38°C. His laboratory data were within the normal limit except for relatively low albumin (2.6 g/dl) and still low lymphocyte count (470/μl). He looks lethargy. We thought this is due to long-term hospitalization; thus, we transferred him to another hospital for further rehabilitation. However, he returned to our hospital 4 days later for pleural effusion of unknown etiology. He had massive pleural effusion in his left chest, and his ascites had not diminished. Thoracentesis was performed on admission, and the pleural fluid was positive for adenosine deaminase (ADA) as 128 IU/l. Ascites were also positive for ADS as 156.6 IU/l. QuantiFERON was positive, and mycobacterial culture of gastric secretions revealed *Mycobacterium tuberculosis*. Sputum culture and Gaffky staining were negative. Repeated stool microscopy was negative for *Strongyloides*. The patient transferred to another hospital for tuberculosis treatment.

We encountered a case of *Strongyloides* hyperinfection, which is rarely seen in Japan. This patient also developed a tuberculosis infection after successful treatment of his strongyloidiasis. Although the relationship between these two infections is not apparent, there may be an association because of the unique characteristics of these infections.

Strongyloidiasis is endemic in tropical and subtropical regions. In Japan, the Okinawa prefecture, which is in the southern islands, is the only endemic region [1]. Because our patient had never been to the Okinawa prefecture, we believe he became infected in Nepal. *Strongyloides* can cause acute, chronic, hyper, and disseminated infection. *Strongyloides* hyperinfection occurs due to the unique life cycle of the *Strongyloides stercoralis*. Their eggs are embedded in the intestinal mucosal epithelium, and non-infectious rhabditiform larvae hatch into the intestinal lumen. The rhabditiform larvae become free-living adults when secreted in feces into the soil. They can develop to infectious filariform larvae, at which point they can penetrate human skin and invade the intestine via the bloodstream [2]. *Strongyloides* hyperinfection is an accelerated autoinfection in which increasing numbers of infectious filariform larvae are produced. Such hyperinfections can occur when the host becomes immunosuppressed, such as with steroid use or HTLV-1 infection [2]. These infections can occur even 57 years after chronic infection [3]. In chronic infection, more than 50% of patients are asymptomatic [4]. Our patient had no symptoms and had not been to an endemic area for 2 years; therefore, we believe this patient had been chronically infected since he was in Nepal and became hyperinfected in Japan. Although this patient does not have history of steroid use or HTLV-1 infection. The patient had worked so hard that he could not eat well. Malnutrition induced decreased number of lymphocyte and subsequently hyperinfected with *Strongyloides*.

Tuberculosis is a re-emerging infection in Japan and is endemic in Nepal. It is an infection that can be re-activated when the host becomes immunosuppressed. There is one previous report in the literature of a co-infection with strongyloidiasis and tuberculosis [5]. The authors concluded that their patient developed tuberculosis because of *Strongyloides* hyperinfection. However, the possibility that the patient developed strongyloidiasis hyperinfection during the course of the tuberculosis infection is not discussed. Similarly, in our patient, it is not clear whether the strongyloidiasis stimulated the tuberculosis re-activation or vice versa. However, it is clear that both *Strongyloides* infection and tuberculosis infection are associated with impaired cellular immunity [6].

Our patient’s lymphocyte count was low, indicating that he was at risk of infection with both strongyloidiasis and tuberculosis. We estimated that malnutrition due to poor diet caused decreased lymphocyte. The decreased lymphocyte caused *Strongyloides* hyperinfection and tuberculosis. We believe *Strongyloides* hyperinfection occurred earlier and subsequently tuberculosis, because there were no signs and symptoms of tuberculosis infection on admission. Regardless, patients with either strongyloidiasis or tuberculosis should be examined carefully for other infections, particularly when the patient has impaired cellular immunity.

## Conclusion

We encountered a unique case in Japan of *Strongyloides* infection in a patient who subsequently was diagnosed with tuberculosis. These infections can therefore present even in non-endemic regions. It is important to consider additional infections when treating a patient with an infection caused by impairment of cellular immunity.

## Consent

Written informed consent was obtained from the patient for publication of this case report and any accompanying images. A copy of the written consent is available for review by the Editor-in-Chief of this journal.
